# Characteristics of culprit intracranial plaque without substantial stenosis in ischemic stroke using three-dimensional high-resolution vessel wall magnetic resonance imaging

**DOI:** 10.3389/fnins.2023.1160018

**Published:** 2023-03-23

**Authors:** Xia Tian, Zhang Shi, Zhen Wang, Bing Xu, Wen-Jia Peng, Xue-Feng Zhang, Qi Liu, Shi-Yue Chen, Bing Tian, Jian-Ping Lu, Cheng-Wei Shao

**Affiliations:** ^1^Department of Radiology, Changhai Hospital, Naval Medical University, Shanghai, China; ^2^Department of Radiology, Zhongshan Hospital, Fudan University, Shanghai, China

**Keywords:** 3D high-resolution magnetic resonance imaging, intracranial non-stenotic atherosclerotic plaques, ischemic stroke, histogram features, diagnostic efficacy

## Abstract

**Background and aims:**

We aim to analyze the difference in quantitative features between culprit and non-culprit intracranial plaque without substantial stenosis using three-dimensional high-resolution vessel wall MRI (3D hr-vw-MRI).

**Methods:**

The patients with cerebral ischemic symptoms of the unilateral anterior circulation were recruited who had non-stenotic intracranial atherosclerosis (<50%) confirmed by computed tomographic angiographic (CTA) or magnetic resonance angiography (MRA). All patients underwent 3D hr-vw MRI within 1 month after symptom onset. 3D hr-vw-MRI characteristics, including wall thickness, plaque burden, enhancement ratio, plaque volume and intraplaque hemorrhage, and histogram features were analyzed based on T2-, precontrast T1-, and post-contrast T1-weighted images. Univariate and multivariate logistic regression analysis were used to identify key determinates differentiating culprit and non-culprit plaques and to calculate the odds ratios (ORs) with 95% confidence intervals (CIs).

**Results:**

A total of 150 plaques were identified, of which 133 plaques (97 culprit and 36 non-culprit) were in the middle cerebral artery, three plaques (all culprit) were in the anterior cerebral artery (ACA) and 14 (11 culprit and three non-culprit) were in the internal carotid artery (ICA). Of all the quantitative parameters analyzed, plaque volume, maximum wall thickness, minimum wall thickness, plaque burden, enhancement ratio, coefficient of variation of the most stenotic site, enhancement ratio of whole culprit plaque in culprit plaques were significantly higher than those in non-culprit plaques. Multivariate logistic regression analysis found that plaque volume [OR, 1.527 (95% CI, 1.231–1.894); *P* < 0.001] and enhancement ratio of whole plaque [OR, 1.095 (95% CI, 1.021–1.175); *P* = 0.011] were significantly associated with culprit plaque. The combination of the two features obtained a better diagnostic efficacy for culprit plaque with sensitivity and specificity (0.910 and 0.897, respectively) than each of the two parameters alone.

**Conclusion:**

3D hr-vw MRI features of intracranial atherosclerotic plaques provided potential values over prediction of ischemic stroke patients with non-stenotic arteries. The plaque volume and enhancement ratio of whole plaque of stenosis site were found to be effective predictive parameters.

## Introduction

Ischemic stroke (IS), defined as sudden neurologic dysfunction caused by focal brain ischemia with imaging evidence of infarction, is a leading cause of death and disability in the world (Mendelson and Prabhakaran, 2021). Nowadays, intracranial atherosclerosis (ICAS) has been recognized as the main cause of IS, accounting for up to 40% of stroke in Asian (Wong, 2006). Treatment strategies for IS suggested by current practice guidelines are mainly based on the degree of luminal stenosis within major intracranial arteries, and there should be worthy of attention in patients with the stenosis more than 50%) (Warfarin-Aspirin Symptomatic Intracranial Disease [WASID] Trial Investigators, 2003; Teng et al., 2016; Zhu et al., 2018; Huang et al., 2019). However, recent autopsy studies have proved that a fair proportion of fatal ischemic stroke may be caused by atherosclerotic plaque in the absence of severe luminal stenosis (Mazighi et al., 2008; Bodle et al., 2013). In general, these patients with intracranial stenosis less than 50%, also called as non-stenotic patients, always have clinical symptoms, who still should be treated actively. Although luminal stenosis could be evaluated by digital subtraction angiography (DSA) or non-invasive methods including computed tomographic angiographic (CTA) and magnetic resonance angiography (MRA), it can’t be noted the vessel wall, where the atherosclerotic plaque originates (Wang et al., 2019). Therefore, the role of non-stenotic intracranial plaque was probably underestimated in the development of IS.

Three-dimensional high-resolution vessel wall MRI (3D hr-vw-MRI) provides a novel method to directly assess the intracranial artery wall, which has been increasingly used to study the atherosclerotic plaque, especially the non-stenotic intracranial plaque (Zhang et al., 2015; Yang et al., 2016). Compared with previous 2D hr-vw-MRI, this new sequence has the unique advantages of whole-brain coverage, higher spatial resolution, higher signal-to-noise ratio, and the ability of isotropic multi-planar reconstruction (Zhu et al., 2016). Many previous studies have shown a promising application of 3D hr-vw MRI for visualizing the intracranial wall and identifying high risk features in patients with intracranial atherosclerosis (Lin et al., 2021; Tian et al., 2021; Zhu et al., 2021). But most of these intracranial vessel wall imaging studies mainly focused on the high-grade stenosis (>50%) (Turan et al., 2011; Ryu et al., 2014; Wu et al., 2018). Recently, the potential of 3D hr-vw-MRI in intracranial plaque with low-grade stenosis (<50%) has just been preliminarily studied (Lu S. S. et al., 2018; Yang et al., 2021). Unfortunately, they have mostly focused on lesion morphological and compositional features. The distribution of signal intensity, which is defined as the number of pixels with signal intensity in a certain categorical range, might also provide clinically relevant information, such as histogram features in atherosclerotic plaque (Shi et al., 2020). However, the histogram analysis of intracranial atherosclerotic plaques without substantial stenosis was not involved and risk factors for IS among vessel wall imaging features deserves further exploration.

In the present study, we aim to evaluate the morphology and histogram analysis of intracranial plaque without substantial stenosis in IS patients using 3D hr-vw-MRI, in purpose of determining plaque features associated with recent cerebrovascular events.

## Materials and methods

### Study population

The study was approved by the local Institutional Review Board (CHEC2018-092) and written informed consent was obtained from all included patients.

Patients who presented between January 2019 and August 2021 were recruited. The demographics including age, sex, history of smoking, hypertension, diabetes, hyperlipidemia, and coronary heart disease were recorded. The National Institute of Health Stroke Scale (NIHSS) on admission were also documented.

The patients should be included as follows: (i) patients with cerebral ischemic symptoms in the unilateral anterior circulation; (ii) the degree of intracranial artery stenosis <50% measured by CTA or MRA; (iii) underwent 3D hr-vw MRI within 1 month of ischemic events; and (iv) patients aged >18 years old.

The exclusion criteria were as follows: (i) non-atherosclerotic intracranial artery disease, such as aneurysm, dissection, arteritis, or moyamoya disease; (ii) suspected cardiogenic stroke; (iii) extracranial artery stenosis ≥50% confirmed by CTA, MRA or ultrasound; (iv) aortic arch atherosclerotic plaque with ulcer or aortic arch wall thickness ≥4 mm; (v) coagulation dysfunction, heart or respiratory failure; (vi) intracranial hemorrhage; (vii) severe disturbance of consciousness; (viii) poor image quality; and (ix) contraindications to MRI.

### MRI protocol

All MRI and MRA examinations were performed with a 3.0T whole-body MRI system (Skyra, Siemens Healthcare, Erlangen, Germany) using a 20-channel head/neck coil. A standard protocol was applied to acquire high-resolution intracranial vessel wall imaging that included 3D time-of-flight (TOF) MRA sequences and 3D hr-vw MRI {including T2-weighted imaging (T2WI) and T1-weighted imaging (T1WI) [3D Sampling Perfection with Application optimized Contrasts using different flip angle Evolution (SPACE) sequence]}. The 3D TOF MRA was scanned in the axial plane by using the following parameters: repetition time (TR)/echo time (TE) = 21/3.43 ms, field of view (FOV) = 181 × 200 mm^2^, slice thickness = 0.7 mm, number of slices = 144, matrix = 331 × 384 and scan time = 4.58 min. 3D hr-vw MRI was acquired in the sagittal plane with a spatially non-selective excitation. The main parameters of 3D hr-vw MRI were as follows: (1) T2-weighted imaging: isotropic resolution = 0.5 mm, FOV = 256 × 160 mm^2^, matrix = 320 × 256, number of slices = 240, echo train length (ETL) = 100, TR/TE = 1,500/150 ms, and scan time = 6.27 min; (2) precontrast T1-weighted imaging: isotropic resolution = 0.5 mm, FOV = 256 × 160 mm^2^, matrix = 320 × 256, number of slices = 240, echo train length (ETL) = 60, TR/TE = 800/17 ms and scan time = 6.45 min. (3) Postcontrast T1-weighted imaging was repeated 7 min after intravenous administration of gadolinium (Gadovist; Bayer Schering Pharma) at a dose of 1.5 mmol/kg.

### Image analysis

Quantitative imaging features were measured on 3D hr-vw-MRI by two neuroradiologists (one with over 11 years of experience and the other with over 12 years of experience) who were blinded to the patient information. Non-stenotic atherosclerotic plaque was defined as eccentric wall thickening without substantial lumen stenosis (<50%) identified on high-resolution magnetic resonance images. The culprit plaque was defined as a lesion arising on the ipsilateral side within the same vascular territory to a hyperintensity signal on the MRI DWI images in patients with accompanying clinical symptoms. A lesion was deemed a non-culprit plaque when it occurred in asymptomatic patients (Shi et al., 2020). If multiple plaques were present in the same vascular territory, the lesion with the most degree of stenosis was chosen for analysis. If there are any different opinions in plaque classification, consensus can be reached through consultation.

Image analysis was performed using VesselMass software with 3-dimensional multiplanar reformation (Leiden University Medical Center, Netherlands). If the slice is not perpendicular to the direction of the long axis of the artery, reconstruction of the cross-sectional image with multi-planner reformation was performed. The lumen and outer wall boundary of the most stenotic lesion, proximal normal lesion and distal normal lesion were firstly manually segmented on T2WI. Then, the lumen and outer wall boundary of all lesions of the whole plaque were semi-automatically segmented on T2WI. Finally, the drawn contours of T2WI were copied to the precontrast T1WI and postcontrast T1WI sequences corresponding to the same lesions.

The quantitative parameters could be obtained automatically by the software: (a). the most stenotic lesion: [morphology parameters: D_*s*_, minimum lumen area (MLA), vessel area (VA_*s*_), wall area (WA_*s*_), maximum wall thickness (Wall_*max*_ thickness), minimum wall thickness (Wall_*min*_ thickness), S_*plaque precontrast*_, S_*plaque postcontrast*_, S_*gray matter precontrast*_ and S_*gray matter postcontrast*_]; (b). proximal and distal normal lesion: [D_*proximal*_, D_*distal*_, vessel area of proximal (VA_*proximal*_) and distal (VA_*distal*_) normal lesion]; (c). whole plaque: plaque volume, S_*whole plaque precontrast*_ and S_*whole plaque postcontrast*_. The following indexes are calculated from the above parameters:


Stenosis=(D-nD)s/D×n100%[D=n(D+proximalD)distal/2].

Plaqueburden(PB)=(1-MLA/VA)s×100%.

Remodelingratio(RR)=VA/sVA[VA=n(VA+proximalVA)distal/2]n.

Eccentricity⁢index⁢(EI)=(Wall⁢thicknessmax-Wall⁢thicknessmin)/Wall⁢thicknessmax.

Enhancementratio=(stenosis)[(S/plaque⁢postcontrastS)gray⁢matter⁢postcontrast/(S/plaque⁢precontrastS)gray⁢matter⁢precontrast-1]×100%.

Enhancementratio=(whole⁢plaque)[(S/whole⁢plaque⁢postcontrastS)gray⁢matter⁢postcontrast/(S/whole⁢plaque⁢precontrastS)gray⁢matter⁢precontrast-1]×100%.

Intraplaque hemorrhage (IPH) was identified if the ratio of signal intensity in a region within the plaque to that in one of the adjacent muscles on precontrast T1WI images was more than 150%.

The quantitative histogram parameters on intracranial atherosclerotic plaques were measured on T1WI image the most stenotic lesion as described in previous study (Shi et al., 2020), which included mean, median, standard deviation (SD), maximum value, minimum value, entropy. The coefficient of variation (CV) was defined as CV = SD/mean.

To assess the reproducibility of the image analysis, thirty patients were additionally randomly selected and all the quantitative parameters of the images were measured by another neuroradiologist (with 12 years of experience) using the same software.

### Statistical analysis

All statistical analyses were performed using SPSS version 20 (IBM, Armonk, NY, USA). Normality testing with Shapiro-Wilk’s test was performed to assess the variable distribution. Continuous variables were expressed as the means ± standard deviation (SD) and were compared with *t*-test or Mann-Whitney U-test. Categorical data were described as counts or percentages, which were compared using *χ^2^* or Fisher’s exact test. Multivariable logistic regression analysis was performed, which included variables with *P* < 0.05 in univariate tests, to identify different imaging and clinical parameters associated with plaque type. The odds ratios (ORs) with 95% confidence intervals (CIs) were calculated by logistic regression model. The diagnostic performance of different single index and combination index were examined using receiver operating characteristic (ROC) curves and area under curve (AUC) values. The inter-reader agreement was evaluated using the Bland-Altman analysis and intraclass coefficient (ICC) value. ICC value >0.75 indicates a preferable consistency between the two readers. *P*-value < 0.05 was considered as statistically significant.

## Results

### Patients and plaques

From an initial cohort of 209 patients, 59 cases were excluded because of extracranial artery stenosis ≥50% (*n* = 7), aneurysm (*n* = 15), vasculitis (*n* = 5), moyamoya disease (*n* = 3), intracranial artery dissection (*n* = 10), suspected cardiogenic stroke (*n* = 5), aortic arch atherosclerotic plaque ulcer or aortic arch wall thickness ≥4 mm (*n* = 5), intracranial hemorrhage (*n* = 2), severe disturbance of consciousness (*n* = 2) and poor image quality (*n* = 5). Finally, 150 patients (113 men and 37 women) were recruited, including 111 culprit plaques from ischemic stroke patients and 39 non-culprit ones form asymptomatic patients. Of these lesions, 133 plaques were located at middle cerebral artery (MCA), 3 at anterior cerebral artery (ACA) and 14 at internal carotid artery (ICA). In the culprit plaque group, 87.4% (97/111) of plaques were located at MCA, 2.7% (3/111) at ACA and 9.9% (11/111) at ICA, while 92.3% (36/39) plaques were located at MCA and 7.7% (3/39) were at ICA for the non-culprit plaque group. The patient demographics and plaque location were shown in Table 1. There were no significant differences in the age, sex, smoking, drinking, diabetes mellitus, hypertension, and coronary heart disease between the two groups (all *P* > 0.05).

**TABLE 1:** Demographics and plaque location between culprit and non-culprit plaques.

	Culprit group (*n* = 111)	Non-cluprit group (*n* = 39)	*P*-value
Age (year)	58.5 ± 12.6	58.9 ± 10.6	0.876
Sex (%)	–	–	0.959
Male	83 (74.8%)	30 (76.9%)	-
Female	28 (25.2%)	9 (23.1%)	-
Smoking (%)	48 (43.2%)	10 (25.6%)	0.080
Alcohol (%)	28 (25.2%)	7 (17.9%)	0.481
Diabetes (%)	31 (27.9%)	9 (23.1%)	0.705
Hypertension (%)	60 (54.1%)	16 (41.0%)	0.225
Coronary artery disease (%)	2 (1.8%)	0 (0%)	0.974
Hyperlipidemia (%)	40 (36.0%)	3 (7.7%)	0.002*
NIHSS score	3 (0–18)	0 (0–6)	<0.001*
Plaque location (%)	–	–	0.526
MCA	97 (87.4%)	36 (92.3%)	-
ICA	3 (2.7%)	0 (0%)	-
BA	11 (9.9%)	3 (7.7%)	-

MCA, middle cerebral artery; ICA, internal carotid artery; BA, basilar artery.

*P < 0.05; **P < 0.01; ***P < 0.001.

### hr-vw MRI characteristics and histogram features of ICAS

**FIGURE 1:**
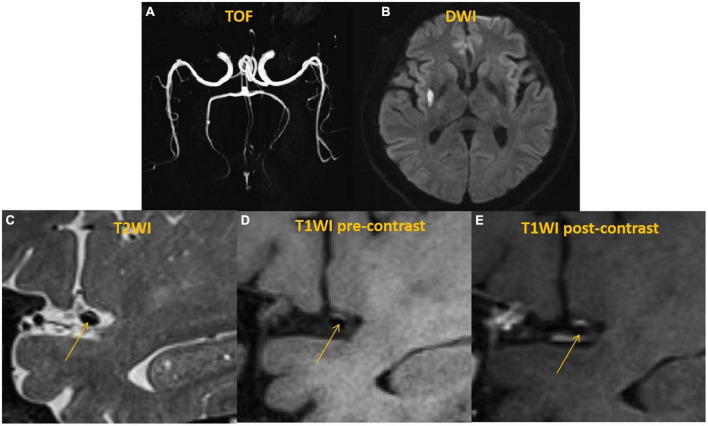
A 59 years-old man with acute ischemic stroke in the right basal ganglia. Time-of-flight (TOF)-magnetic resonance angiography (MRA) **(A)** demonstrated the stenosis located on the right middle cerebral artery (MCA). Diffusion weighted imaging (DWI) **(B)** showed a sheet acute infarcts in the right basal ganglia. High-resolution vessel wall MRI (hr-vw MRI) including T2-weighted image **(C)**, pre-contrast T1-weighted image **(D)** and post-contrast T1-weighted image **(E)** visualized the plaque on the superior side of the right MCA. The plaque showed isointense to hyperintense on T2-weighted image **(C)** and pre-contrast T1-weighted image **(D)** and moderate enhancement on post-contrast T1-weighted image **(E)**.

**FIGURE 2:**
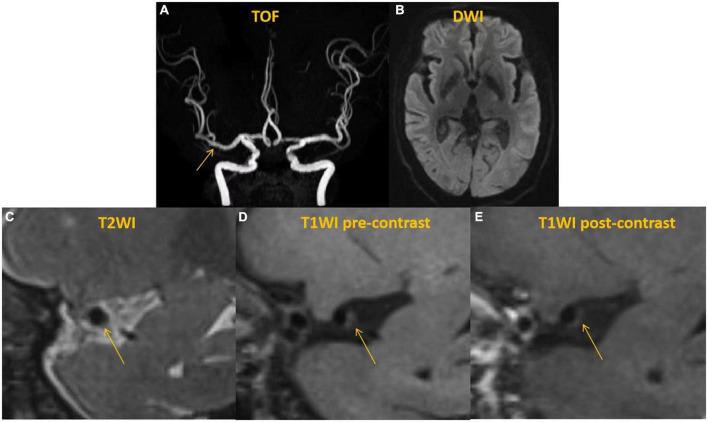
A 62 years-old man without any neuro symptom. Time-of-flight (TOF)-magnetic resonance angiography (MRA) **(A)** demonstrated a normal appearance. DWI **(B)** showed no acute infarct with the anterior circulation territory. High-resolution vessel wall MRI (hr-vw MRI) including T2-weighted image **(C)**, pre-contrast T1-weighted image **(D)** and post-contrast T1-weighted image **(E)** visualized the plaque on the posterior and inferior side of the right middle cerebral artery (MCA). The plaque showed isointense on T2-weighted image **(C)** and pre-contrast T1-weighted image **(D)** and no enhancement on post-contrast T1-weighted image **(E)**.

Images from representative case of culprit plaque and non-culprit plaque were shown in Figures 1, 2. As shown in Table 2, there were significant differences in plaque volume, wall_*max*_ thickness, wall_*min*_ thickness, plaque burden, enhancement ratio of stenosis stie and whole plaque between culprit plaques and non-culprit ones (all *P* < 0.05). Furthermore, in histogram analysis, it illustrated that CV was significantly different between culprit and non-culprit lesions (*P* = 0.043). However, no significant differences were observed in other histogram parameters between the two plaque types.

**TABLE 2:** Comparison of high-resolution vessel wall magnetic resonance imaging (hr-vw MRI) characteristics and histogram features between culprit and non-culprit plaques.

	Culprit group (*n* = 111)	Noncluprit group (*n* = 39)	*P*-value
**hr-vw MRI**			
MLA (mm^2^)	5.00 ± 2.33	54.28 ± 1.61	0.075
Wall_max_ thickness (mm)	1.65 ± 0.43	1.23 ± 0.24	<0.001***
Wall_min_ thickness (mm)	0.37 ± 0.17	0.31 ± 0.09	<0.001*
Stenosis (%)	16.92 ± 9.13	14.34 ± 8.85	0.128
Plaque burden (%)	69.95 ± 8.69	62.55 ± 5.19	0.003**
Remodeling index (%)	104.73 ± 27.85	97.00 ± 22.73	0.121
Eccentricity index (%)	77.16 ± 9.68	74.98 ± 7.22	0.202
Enhancement ratio_(stenosis site)_ (%)	39.20 ± 20.60	15.58 ± 9.54	<0.001***
Enhancement ratio_(whole plaque)_ (%)	42.44 ± 20.79	16.66 ± 9.68	<0.001***
Plaque volume (mm^3^)	37.22 ± 11.46	17.77 ± 6.02	<0.001***
Intraplaque hemorrhage (%)	13 (11.7%)	1 (2.6%)	0.171
**Histogram (pre-T1)**			
Mean	119.15 ± 36.49	123.75 ± 32.00	0.486
SD	34.39 ± 12.58	32.19 ± 10.48	0.328
Median	118.13 ± 36.96	120.72 ± 37.46	0.708
Minimum value	43.80 ± 23.37	46.61 ± 20.83	0.508
Maximum value	196.35 ± 55.56	194.55 ± 48.88	0.858
CV	0.29 ± 0.09	0.26 ± 0.06	0.043*
Entropy	6.62 ± 4.99	6.51 ± 0.37	0.187

MLA, minimum lumen area; SD, standard deviation; CV, coefficient of variation.

*P < 0.05; **P < 0.01; ***P < 0.001.

### Univariate and multivariate logistic regression analysis

**FIGURE 3:**
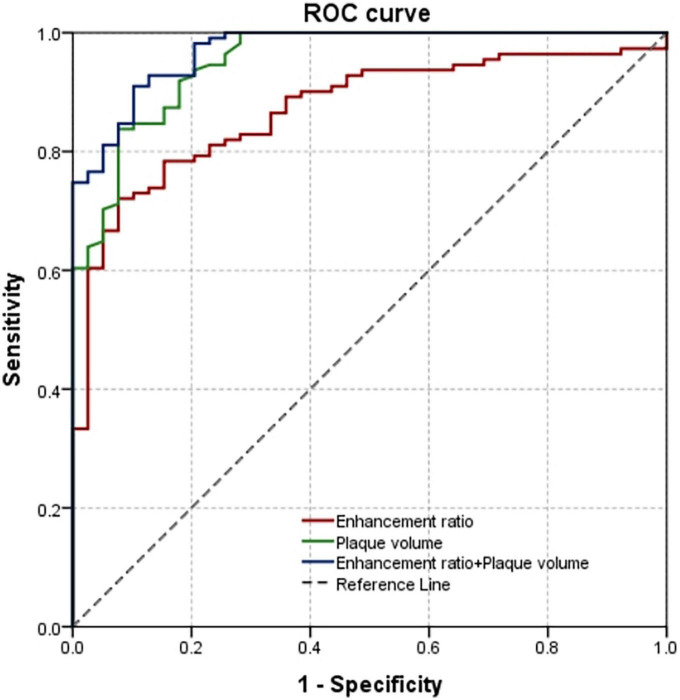
Receiver operating characteristic (ROC) curve on the basis of multivariable logistic regression to differentiate culprit from non-culprit plaques.

Potential predictive parameters listed in Tables 1, 2 with *P* < 0.05 were included in the regression analyses (Table 3). It was shown, in the univariate logistic regression, that hyperlipidemia, NIHSS score, plaque volume, plaque burden, enhancement ratio_(whole plaque)_ and CV in culprit plaque group were significantly higher than those in non-culprit plaque group. Multiple logistic regression analysis showed that plaque volume [OR, 1.527 (95% CI, 1.231–1.894); *P* < 0.001] and enhancement ratio_(*whole plaque*)_ [OR, 1.095 (95% CI, 1.021–1.175); *P* = 0.011] were independent factors for culprit plaque after adjusting for confounding factors such as wall_*max*_ thickness, wall_*min*_ thickness, enhancement ratio of stenosis stie (Table 3). The AUC values of plaque volume and enhancement ratio_(whole plaque)_ were 0.952 (95% CI, 0.918–0.987) and 0.870 (95% CI, 0.811–0.929), respectively. The combination of these two features improved the AUC to 0.970 (95% CI, 0.946–0.994) with sensitivity and specificity being 0.910 and 0.897, respectively (Figure 3).

**TABLE 3:** Univariate and multivariate analyses of the characteristics and histogram features associated with culprit plaques.

	Univariate analysis, mean ± SD or *N* (%) or M (Q_L_, Q_U_)			Multivariate analysis		
	**Culprit group (*n* = 111)**	**Non-cluprit group (*n* = 39)**	***P*** **-value**	**OR**	**95% CI**	***P*** **-value**
Hyperlipidemia (%)	40 (36.0%)	3 (7.7%)	0.003	0.102	0.010–0.998	0.050
NIHSS score	3 (0–18)	0 (0–6)	<0.001	1.759	0.933–3.318	0.081
Plaque volume (mm^3^)	37.22 ± 11.46	17.77 ± 6.02	<0.001	1.527	1.231–1.894	<0.001***
Plaque burden (%)	69.95 ± 8.69	62.55 ± 5.19	0.005	0.901	0.765–1.062	0.214
Enhancement ratio_(whole plaque)_ (%)	42.44 ± 20.79	16.66 ± 9.68	<0.001	1.095	1.021–1.175	0.011*
CV	0.29 ± 0.09	0.26 ± 0.06	0.043	1.031	0.930–1.143	0.562

CV, coefficient of variation.

*P < 0.05; ***P < 0.001.

### Reproducibility of quantitative measurements

There was good inter-reader agreement between the two readers for the quantitative measurements of morphological characteristics and histogram features on 3D hr-vw MRI. All the ICC values of the parameters involved in this study were more than 0.80, as shown in Supplementary Table 1.

## Discussion

Our present study explored the characteristics of non-stenotic intracranial plaques in patients with ischemic stroke using 3D hr-vw MRI. The findings showed that plaque volume, wall thickness, plaque burden, enhancement ratio, and one histogram parameter (CV) were significantly different between culprit and non-culprit lesions. Furthermore, multivariate logistic regression analysis demonstrated that plaque volume and enhancement ratio were significantly associated with culprit plaque. And the combination of two parameters could improve the predictive power significantly.

Up to now, the most studies of intracranial arterial wall imaging focus on the plaque with moderate to severe degree of stenosis (>50%) (Teng et al., 2016; Zhu et al., 2018; Huang et al., 2019). The analysis of vessel wall imaging characteristics in plaque without substantial stenosis (<50%) has been reported in several researches (Shi et al., 2021; Yang et al., 2021). In this study, we found that plaque volume was an independent risk factor of culprit plaque in non-stenotic intracranial artery. Teng et al. (2016) reported that culprit lesions exhibit a greater plaque volume than non-culprit controls in MCA with different degrees of stenosis using 2D sequences. Shi et al. (2021) also demonstrated that plaque volume was significantly associated with culprit lesions in MCA and basilar artery with mild luminal stenosis. However, none of those studies showed that plaque volume was an independent risk factor of culprit lesions. It was probably because that those studies involved vessels with different degrees of stenosis and in different areas (including anterior and posterior circulation), which might cause various confounding factors. Nevertheless, one study also found that culprit plaque volume were independently associated with recurrent stroke (Wu et al., 2022). Thus, it should be noted that “plaque volume” is distinct from “plaque burden”: plaque volume is the accumulation of the area in each layer of the whole plaque; plaque burden is the percentage of plaque area in the vessel area in the narrowest layer (single layer) (Lu M. et al., 2018; Wu et al., 2022). Resulted of the positive remodeling effect, the plaque burden on the narrowest layer may not be the largest among the whole plaque layers. However, plaque volume can objectively reflect the overall plaque burden and more comprehensively evaluate the condition of lumen and vessel wall.

Moreover, we found that enhancement ratio of the whole plaque in culprit plaque group was significantly higher than that in non-culprit plaque group, whereas no significant difference of that located on the most stenotic lesion. Previous studies on intracranial hr-vw-MRI have shown that plaque enhancement is a significant marker that is strongly linked to culprit lesions and clinical outcomes. However, our findings do not entirely align with these studies (Lu et al., 2021; Zhang et al., 2021; Zhao et al., 2021), as we discovered that the enhancement ratio was considerably higher in culprit lesions than in non-culprit ones, rather than in most stenotic plaques across the entire plaque. It could be interpreted with the following considerations. First, we focused on the non-stenotic intracranial plaque, which has a low-grade stenosis (<50%) and relatively small plaque volume. The enhancement ratio of the whole plaque is more objective in assessment of vessel wall than the enhancement ratio of the most stenotic lesion, which may more likely to bring out positive results on differentiating the plaque type. Second, the layer with the most enhancement ratio is not always the most stenotic lesion. It should be noted that further multiple logistic regression analyses in this study showed that enhancement ratio of whole plaque was an independent factor associated with the culprit plaque in symptomatic patients suffered by non-stenotic intracranial atherosclerosis.

Recently, the histogram analysis of the plaque in ICAS has become a novel research area. Our group has proved that the histogram analysis could better improve the accuracy for the lesion type differentiation compared with conventional hr-vw MRI-defined parameters (Shi et al., 2020). In our current study, the histogram analysis was calculated by T1-weighted MR images from 3D sequence, and the finding showed a significant difference in CV between culprit and non-culprit lesions in intracranial artery without substantial stenosis. However, it was excluded by multiple logistic regression analysis, which was different from the previous study (Shi et al., 2020). Our present study focused on the non-stenotic intracranial plaque, in which the MR image analysis of histogram features was more difficult. Besides, the plaques with poor homogeneity may have more smaller volume, in which the role of the plaque homogeneity could be underestimated. Moreover, the sample of this study was limited. Considering the great value of histogram characteristics in differentiating atherosclerotic plaques, it is still worthy of deep exploration in further studies with larger samples.

There are some limitations in this study. First, this is a single center study base on Chinese patients. Second, although the 3D volume scanning sequence reduces the error caused by partial volume effect, the relative thin vessel diameter and small plaque volume in intracranial artery are tremendous challenges for the analysis of compositional characteristics on vessel wall MR imaging. Third, for asymptomatic patients, many clinicians do not routinely perform three-dimensional high-resolution vessel wall MRI because of their possible normal CTA or MRA performance. Thus, the number of cases of non-culprit plaques is low. Last, it is extremely difficult to obtain the tissue of intracranial atherosclerotic plaque in clinic. The confirmation of IPH, lipid composition and enhancement characteristics of the plaque by histopathology is not able to achieve.

In conclusion, this study indicated that plaque volume and maximum wall thickness in low-grade stenosis (<50%) were significantly associated with the type of intracranial atherosclerotic plaque, which can be potentially used to identify lesions responsible for acute ischemic stroke in ICAS patients. These imaging characteristics might serve to differentiate culprit plaque beyond luminal stenosis and benefit patients from early interventions to avoid downstream cerebrovascular events.

## Data availability statement

The raw data supporting the conclusions of this article will be made available by the authors, without undue reservation.

## Ethics statement

The studies involving human participants were reviewed and approved by Ethics Committee of Changhai Hospital. The patients/participants provided their written informed consent to participate in this study.

## Author contributions

XT, ZS, ZW, BX, S-YC, and BT: protocol/project development. BX, ZS, W-JP, X-FZ, and QL: data collection or management. XT, J-PL, and C-WS: data analysis. All authors contributed to the article and approved the submitted version.

## References

[B1] Bodle J. D. Feldmann E. Swartz R. H. Rumboldt Z. Brown T. Turan T. N. (2013). High-resolution magnetic resonance imaging: An emerging tool for evaluating intracranial arterial disease. *Stroke* 44 287–292. 10.1161/STROKEAHA.112.664680 23204050 PMC3556720

[B2] Huang J. Jiao S. Zhao X. Zhang J. Zhang C. Chen M. (2019). Characteristics of patients with enhancing intracranial atherosclerosis and association between plaque enhancement and recent cerebrovascular ischemic events: A high-resolution magnetic resonance imaging study. *Acta Radiol.* 60 1301–1307. 10.1177/0284185118822645 30650984

[B3] Lin G. Song J. Fu N. Huang X. Lu H. (2021). Quantitative and qualitative analysis of atherosclerotic stenosis in the middle cerebral artery using high-resolution magnetic resonance imaging. *Can. Assoc. Radiol. J.* 72 783–788. 10.1177/0846537120961312 33023323

[B4] Lu M. Peng P. Cui Y. Qiao H. Li D. Cai J. (2018). Association of progression of carotid artery wall volume and recurrent transient ischemic attack or stroke: A magnetic resonance imaging study. *Stroke* 49 614–620. 10.1161/STROKEAHA.117.019422 29382804

[B5] Lu S. S. Ge S. Su C. Q. Xie J. Shi H. B. Hong X. N. (2018). Plaque distribution and characteristics in low-grade middle cerebral artery stenosis and its clinical relevance: A 3-dimensional high-resolution magnetic resonance imaging study. *J. Stroke Cerebrovasc. Dis.* 27 2243–2249. 10.1016/j.jstrokecerebrovasdis.2018.04.010 29752069

[B6] Lu Y. Ye M. Zhao J. Diao S. Li T. Ding D. (2021). Gadolinium enhancement of atherosclerotic plaque in the intracranial artery. *Neurol. Res.* 43 1040–1049. 10.1080/01616412.2021.1949682 34229565

[B7] Mazighi M. Labreuche J. Gongora-Rivera F. Duyckaerts C. Hauw J. Amarenco P. (2008). Autopsy prevalence of intracranial atherosclerosis in patients with fatal stroke. *Stroke* 39 1142–1147. 10.1161/STROKEAHA.107.496513 18309170

[B8] Mendelson S. Prabhakaran S. (2021). Diagnosis and management of transient ischemic attack and acute ischemic stroke: A review. *JAMA* 325 1088–1098. 10.1001/jama.2020.26867 33724327

[B9] Ryu C. Jahng G. Shin H. (2014). Gadolinium enhancement of atherosclerotic plaque in the middle cerebral artery: Relation to symptoms and degree of stenosis. *Am. J. Neuroradiol.* 35 2306–2310. 10.3174/ajnr.A4038 25012673 PMC7965297

[B10] Shi Z. Li J. Zhao M. Peng W. Meddings Z. Jiang T. (2020). Quantitative histogram analysis on intracranial atherosclerotic plaques: A high-resolution magnetic resonance imaging study. *Stroke* 51 2161–2169. 10.1161/STROKEAHA.120.029062 32568660 PMC7306260

[B11] Shi Z. Zhao M. Li J. Meddings Z. Shi Y. Jiang T. (2021). Association of hypertension with both occurrence and outcome of symptomatic patients with mild intracranial atherosclerotic stenosis: A prospective higher resolution magnetic resonance imaging study. *J. Magn. Reson. Imaging* 54 76–88. 10.1002/jmri.27516 33694230 PMC8319792

[B12] Teng Z. Peng W. Zhan Q. Zhang X. Liu Q. Chen S. (2016). An assessment on the incremental value of high-resolution magnetic resonance imaging to identify culprit plaques in atherosclerotic disease of the middle cerebral artery. *Eur. Radiol.* 26 2206–2214. 10.1007/s00330-015-4008-5 26376883 PMC4902836

[B13] Tian X. Tian B. Shi Z. Wu X. Peng W. Zhang X. (2021). Assessment of intracranial atherosclerotic plaques using 3D black blood MRI: Comparison with 3D time-of-flight MRA and DSA. *J. Magn. Reson. Imaging* 53 469–478. 10.1002/jmri.27341 32864816

[B14] Turan T. N. Bonilha L. Morgan P. S. Adams R. J. Chimowitz M. I. (2011). Intraplaque hemorrhage in symptomatic intracranial atherosclerotic disease. *J. Neuroimaging* 21 e159–e161. 10.1111/j.1552-6569.2009.00442.x 19909397

[B15] Wang Y. Liu X. Wu X. Degnan A. J. Malhotra A. Zhu C. (2019). Culprit intracranial plaque without substantial stenosis in acute ischemic stroke on vessel wall MRI: A systematic review. *Atherosclerosis* 287 112–121. 10.1016/j.atherosclerosis.2019.06.907 31254918 PMC6707846

[B16] Warfarin-Aspirin Symptomatic Intracranial Disease [WASID] Trial Investigators (2003). Design, progress and challenges of a double-blind trial of warfarin versus aspirin for symptomatic intracranial arterial stenosis. *Neuroepidemiology* 22 106–117. 10.1159/000068744 12656117

[B17] Wong L. (2006). Global burden of intracranial atherosclerosis. *Int. J. Stroke* 1 158–159. 10.1111/j.1747-4949.2006.00045.x 18706036

[B18] Wu F. Song H. Ma Q. Xiao J. Jiang T. Huang X. (2018). Hyperintense plaque on intracranial vessel wall magnetic resonance imaging as a predictor of artery-to-artery embolic infarction. *Stroke* 49 905–911. 10.1161/STROKEAHA.117.020046 29540606 PMC5871589

[B19] Wu G. Wang H. Zhao C. Cao C. Chai C. Huang L. (2022). Large culprit plaque and more intracranial plaques are associated with recurrent stroke: A case-control study using vessel wall imaging. *Am. J. Neuroradiol.* 43 207–215. 10.3174/ajnr.A7402 35058299 PMC8985671

[B20] Yang H. Ji C. Wang H. Lin L. Yuan X. Liu B. (2021). Characterisation of symptomatic intracranial plaque without substantial stenosis using high-resolution vessel wall MRI. *Clin. Radiol.* 76 392.e21–392.e26. 10.1016/j.crad.2021.01.008 33610287

[B21] Yang H. Zhang X. Qin Q. Liu L. Wasserman B. A. Qiao Y. (2016). Improved cerebrospinal fluid suppression for intracranial vessel wall MRI. *J. Magn. Reson. Imaging* 44 665–672. 10.1002/jmri.25211 26950926

[B22] Zhang L. Zhang N. Wu J. Zhang L. Huang Y. Liu X. (2015). High resolution three dimensional intracranial arterial wall imaging at 3T using T1 weighted SPACE. *Magn. Reson. Imaging* 33 1026–1034. 10.1016/j.mri.2015.06.006 26143482

[B23] Zhang X. Chen L. Li S. Shi Z. Tian X. Peng W. (2021). Enhancement characteristics of middle cerebral arterial atherosclerotic plaques over time and their correlation with stroke recurrence. *J. Magn. Reson. Imaging* 53 953–962. 10.1002/jmri.27351 33034113

[B24] Zhao J. Lu Y. Cui J. Ma L. Zhang R. Xu Z. (2021). Characteristics of symptomatic plaque on high-resolution magnetic resonance imaging and its relationship with the occurrence and recurrence of ischemic stroke. *Neurol. Sci.* 42 3605–3613. 10.1007/s10072-021-05457-y 34236554

[B25] Zhu C. Haraldsson H. Tian B. Meisel K. Ko N. Lawton M. (2016). High resolution imaging of the intracranial vessel wall at 3 and 7T using 3D fast spin echo MRI. *MAGMA* 29 559–570. 10.1007/s10334-016-0531-x 26946509

[B26] Zhu C. Tian X. Degnan A. J. Shi Z. Zhang X. Chen L. (2018). Clinical significance of intraplaque hemorrhage in low- and high-grade basilar artery stenosis on high-resolution MRI. *AJNR Am. J. Neuroradiol.* 39 1286–1292. 10.3174/ajnr.A5676 29794236 PMC6039267

[B27] Zhu T. Ren L. Zhang L. Shao Y. Wan L. Li Y. (2021). Comparison of plaque characteristics of small and large subcortical infarctions in the middle cerebral artery territory using high-resolution magnetic resonance vessel wall imaging. *Quant. Imaging Med. Surg.* 11 57–66. 10.21037/qims-20-310 33392011 PMC7719918

