# Supplementary material for: Characteristics of culprit intracranial plaque without substantial stenosis in ischemic stroke using three-dimensional high-resolution vessel wall magnetic resonance imaging
Source: Front Neurosci. 2023 Mar 23;17:1160018. doi: 10.3389/fnins.2023.1160018 (PMC10076565; doi:10.3389/fnins.2023.1160018)
Supplement: Supplementary file 1 [file Table_1.DOCX]

**Table S1. Interreader agreement between the two readers for the quantitative measurements.**

| **Characteristics** | **ICC（95%CI）** |
| --- | --- |
| Plaque volume | 0.971（0.940-0.986） |
| MLA | 0.980（0.959-0.991） |
| Wall_max_ thickness | 0.983（0.966-0.992） |
| Wall_min_ thickness | 0.971（0.940-0.986） |
| Stenosis | 0.995（0.989-0.997） |
| Plaque burden | 0.955（0.907-0.978） |
| Remodeling index | 0.983（0.964-0.992） |
| Eccentricity index | 0.985（0.969-0.993） |
| Enhancemen ratio_(stenosis site)_ | 0.999（0.997-0.999） |
| Enhancemen ratio_(whole plaque)_ | 0.999（0.997-0.999） |
| Mean | 0.980（0.959-0.990） |
| SD | 0.995（0.990-0.998） |
| Median | 0.953（0.904-0.978） |
| Minimum value | 0.998（0.995-0.999） |
| Maximun value | 0.943（0.883-0.972） |
| CV | 0.994（0.986-0.997） |
| Entropy | 0.800（0.622-0.900） |

MLA, minimum lumen area; SD, standard deviation; CV, coefficient of variation
